# The effect of mask use on cross-race face perception: a simultaneous EEG and eye-tracking study

**DOI:** 10.1186/s41235-026-00704-2

**Published:** 2026-01-29

**Authors:** Yueyuan Zheng, Danni Chen, Xiaoqing Hu, Janet Hsiao

**Affiliations:** 1 Division of Social Science, Hong Kong University of Science and Technology, Clearwater Bay, Kowloon, Hong Kong S.A.R.; 2 Department of Psychology, University of Hong Kong, Pokfulam Road 109, Hong Kong Island, Hong Kong S.A.R.; 3 HKU-Shenzhen Institute of Research and Innovation, Shenzhen, China; 4 Department of Computer Science and Engineering, Hong Kong University of Science and Technology, Kowloon, Hong Kong S.A.R.

**Keywords:** Cross-race perception, Mask, Eye-tracking, EEG, Social categorization

## Abstract

**Abstract:**

While people are often experts in perceiving and categorizing faces into meaningful social categories (i.e., race), there are suboptimal scenarios such as mask use that may impair face processing. Here we examined how mask use may differentially impact own- and other-race face processing in social categorization, and the underlying neurocognitive mechanisms using simultaneous eye movement and EEG recording. We found that mask use made participants’ face scanning patterns more eyes-focused and consistent, and reduced the differences in both eye movement pattern and early attention-related ERP component P1 between viewing own- and other-race faces. Moreover, mask use did not change how people categorize biracial morphed faces, or the advantage in categorization speed of other-race faces. These results suggest that when perceiving masked faces, information from the eye region may be sufficient for social categorization, and that race-based social categorizations can be impervious to mask use. Interestingly, we found that when viewing other-race faces, where people have less perceptual expertise, those who show more consistent face scanning patterns have more efficient processing of masked faces. These findings have important implications for cross-race face perception, especially when face perception condition becomes suboptimal.

**Significance statement:**

As mask use has become a common practice in response to respiratory virus outbreaks, it has inadvertently altered both health practices and the complex dynamics of social interaction. In a world that values diversity and cross-racial interactions, understanding how masks influence our cognitive processes during cross-race face perception is not just timely but vital. Given this context, we examined the effect of mask use on race categorization, by systematically investigating eye movement behavior, and neural representations of own versus other-race faces, and how these mask-induced changes are associated with each other. By utilizing simultaneous eye movement and EEG recording, our study reveals that the eye region can significantly influence social categorization, suggesting that race-based categorizations persist even in the presence of masks. Interestingly, we found that for other-race faces with which people have less perceptual expertise, those who adjust to a more consistent face scanning pattern for masked faces have more efficient processing of masked faces. This highlights the importance of individuals’ visual routine adaptability when the viewing condition is not optimal. Though the current research is called by the demand for COVID-19, our findings can be generalized to a broader context and enhance our understanding of human visual and social cognition.

**Supplementary Information:**

The online version contains supplementary material available at 10.1186/s41235-026-00704-2.

## Introduction

Daily social interaction requires efficient processing of others’ faces and social categorization. However, in suboptimal viewing conditions such as when perceiving masked faces, the use of masks can disrupt face processing on various dimensions, including categorizing gender, age, and identity (e.g., Fitousi et al., 2021; Freud et al., 2020; Gülbetekin et al., 2023). Nevertheless, it remains unclear how mask use influences cross-race face perception and social categorization. Here, we aimed to address this research gap by systematically investigating how mask use influences people’s perception and categorization of their own- and other-race faces, including behavioral responses, eye movement pattern and electrophysiological neural activity.

During cross-race face perception, people are better at recognizing their own race than other-race faces (Meissner & Brigham, 2001 for a review) due to subordinate-level individuation experience with own-race faces (Bukach et al., 2012). In contrast, people are faster in categorizing other-race faces than own-race faces without sacrificing accuracy (Ge et al., 2009), reflecting basic-level categorization experience with other-race faces. Examining eye movement patterns during free viewing, both Asian and White individuals more frequently attend to the eye region of other-race faces and to the nose region of their own-race faces (Hu et al., 2014; Liu et al., 2011; Xiao et al., 2014). In addition, race modulates attention-related and face-sensitive event-related brain potentials (ERPs) across different face processing tasks. For example, other-race faces elicited higher P1 amplitudes than own-race faces in face recognition and in gender identification tasks, and higher P2 amplitudes in the face recognition task (Anzures et al., 2022; He et al., 2009). These results suggest that viewing other-race faces enhances attentional allocation as compared with own-race faces, possibly due to their perceived novelty or social salience. Additionally, other-race faces elicited a more negative N170 than own-race faces in face/identity recognition and face learning tasks (Senholzi & Ito, 2013; Wiese & Schweinberger, 2018), reflecting greater perceptual processing demands required to encode the other-race faces. Furthermore, multivariate EEG decoding results suggested that the race-based social categorization effects can emerge as early as 100 ms (Shoura et al., 2024), suggesting that race categorization may be an automatic cognitive process.

Mask use substantially influences human face perception, making people less accurate and slower in face processing (e.g., Fitousi et al., 2021; Gülbetekin et al., 2023). This can be indicated by how mask use affects face-sensitive ERPs: masked faces elicited lower P1/P2 amplitudes and extended N170 latency in gender and emotion face classification tasks (Pesciarelli et al., 2021; Prete et al., 2022), suggesting that mask use may disrupt the face processing. These behavioral and neural changes due to mask occlusion may be associated with changes in eye movement patterns. Indeed, recent research showed that people who adjust their eye movement pattern to be more eyes-focused and consistent were less affected by the mask use in face recognition performance (Hsiao et al., 2022b). Moreover, people who typically adopted an eyes-focused pattern had smaller performance impairments due to mask use in both own-race face and facial expression recognition (Zheng et al., 2023). Nevertheless, it remains unclear how mask use differentially influences cross-race face perception.

To address this research gap, we recruited East Asian participants to complete a race categorization task, and perform an identity one-back task with simultaneous eye-tracking and EEG recording to assess their baseline eye movement patterns and neural representations during free face viewing. We are primarily interested in how mask use would differentially impact own- and other-race face categorization performance. We then examine how mask use would change participants’ eye movement pattern during free viewing of own- and other-race faces and its neural correlates, and how such changes were associated with categorization performance. To quantitatively assess participants’ eye movement patterns, we adopted an Eye Movement analysis with Hidden Markov Models (EMHMM; Chuk et al., 2014). This data-driven, machine learning based approach enables us to comprehensively examine eye movements by taking both the spatial and temporal dimensions of eye movements into account, as compared with traditional summary statistics such as fixation counts in predefined regions of interest (ROIs; e.g., Goldinger et al., 2009; Wu et al., 2012). For EEG activity, we examined the influence of face race and mask use on attention-related ERP component P1 and face-sensitive N170 and P2 (e.g., Pesciarelli et al., 2021; Prete et al., 2022). In addition, we adopted the multivariate EEG decoding analysis to systematically examine how race, individual identity, and mask would influence the neural representations of faces as assessed in decoding accuracy (e.g., Zhou et al., 2022; Żochowska et al., 2022).

We hypothesized that mask use would (1) impair the cross-race face categorization, (2) direct participants’ eye movement pattern more towards the eyes and increase eye movement consistency during free face viewing, and (3) reduce face-sensitive ERP as well as EEG race decoding accuracy.

## Method

### Participants

We preregistered to collect 50 participants, based on previous EMHMM eye movement studies (*N* = 46–60; Zhang et al., 2019; Chuk et al., 2017; Chan et al., 2018), and EEG face decoding studies (*N* = 13–26; Nemrodov et al., 2018; Ambrus et al., 2019; Dobs et al., 2019; Bae, 2021). This sample size was larger than the required sample size determined by the power analysis of 2 × 2 repeated measures ANOVA assuming a medium effect size (f^2^ = .15, power = .80, α = .05) using MorePower (Campbell & Thompson, 2012), which was 48. Considering potential dropout and data exclusion, we recruited 55 Chinese participants (41 females) between 17 to 30 years old (M = 19.91; SD = 2.86) from a local university. Participants had normal or corrected-to-normal vision with no self-reported cognitive disabilities or psychological problems. Participants’ own-race contact was significantly higher than other-race contact, t(54) = 12.46, *p* < .001, d = 1.68, based on self-reported Racial Contact Questionnaire (Hancock & Rhodes, 2008). Out of 55 participants, 7 participants did not complete the social categorization and bias task due to technical problems, resulting in 48 (34 females; 18–29 years old, M = 19.81, SD = 2.66) complete behavioral data. For EEG analyses, 3 participants were excluded due to extensive EEG artifacts (i.e., remaining epochs number lower than 50%), and 3 participants were excluded because of other reasons (e.g., not native Asian, opting out earlier, and data missing), resulting in 49 participants (36 females; 18–29 years old, M = 19.80, SD = 2.64) being retained. 45 participants (32 females) between 18 to 29 years old (M = 19.87; SD = 2.73) had intact data for all measures. All participants gave their written consent and were compensated with course credit or cash.

### Materials and apparatus

The materials of the social categorization and bias task contained face images from 16 monoracial individuals (8 East Asians and 8 Whites) adapted from Sheng and Han (2012). The 8 biracial morphed unmasked face stimuli were generated from these images, with an artificial combination of 50% East Asian and White faces, using the FunMorph software (http://www.funmorph.com/). The stimuli included an equal number of male and female faces for each race category.

The materials of the identity one-back task contained face images from 16 individuals, with 8 White faces from the FACES database (Ebner et al., 2010) and 8 East Asian faces adapted from Zhang et al. (2019). The stimuli included an equal number of male and female faces for each race category. Each individual had a neutral and a happy face. The inclusion of two distinct face images per individual was specifically designed to facilitate later identity decoding analyses (see Methods).

All face stimuli were aligned at the center point between the two eyes and were grey-scaled and then normalized in luminance and spatial frequency distribution using SHINE Toolbox (Willenbockel et al., 2010). Each face image had an unmasked and a masked version. A surgical mask with luminance and spatial frequency distribution matched to the lower half of the corresponding face was added using Adobe Photoshop software to create masked version. A 510 mm × 290 mm monitor with a resolution of 1920 × 1080 pixels and a keyboard were used to collect behavioral responses. The social categorization and bias task was programmed using PsychoPy (Peirce et al., 2019), and the identity one-back task was coded in MATLAB with PsychoToolbox (Kleiner et al., 2007). The face width was around 8° visual angle (following Hsiao & Cottrell, 2008), resembling the size of a real face seen under a normal conversational distance (i.e., 100 cm).

### Design and procedure

Participants performed the identity one-back task and the social categorization and bias task in order. They then completed the racial contact and demographic questionnaires. The whole experiment lasted for approximately 150 min.

#### Social categorization and bias task

Participants categorized 48 faces (32 monoracial, 16 biracial) as Asian or White. Participants pressed button “A” for Asian answers and button “L” for White answers within 1500 ms. We calculated the percentage of own-race responses for biracial morphed faces as the own-race categorization bias. To examine the effect of mask and race on social categorization performance, we conducted a mask (masked vs unmasked) by face race (Asian vs White) repeated-measures ANOVA on accuracy and RT, and a paired-sample t-test between masked and unmasked conditions on the own-race categorization bias, respectively.

#### Identity one-back task

Participants viewed faces freely in sequence and pressed a key when they saw two consecutive same faces. The task had six blocks, consisted of 144 trials each (128 non-target trials: 4 repetitions of 32 face images, 16 target trials). Each individual’s faces were presented in either masked or unmasked condition, and the mask condition of each individual was counterbalanced among participants. Target trials were excluded from analyses. The purpose of the one-back design was to ensure that participants stayed focused and processed facial information during free viewing, as evidenced by their high accuracy on target trials (M = 0.94, SD = 0.09). Each trial started with a drift check, followed by a blank screen lasting for a randomized duration between 800 and 1200 ms, and an 800 ms fixation cross. The face image was presented for 1000 ms. Participants’ eye movements and brain waves were simultaneously recorded.

Mask conditions by face race repeated-measures ANOVAs on the eye movement and neural representation measures were conducted to examine the effect of mask and race on free face viewing. We also calculated the mask effects (unmask-minus-mask condition) consistently for all race categories and measures, and examined the relationship among mask effects in eye movement and neural representation measures in the identity one-back task, and behavioral measures in the social categorization and bias task using Pearson’s correlations. In addition, to examine whether individuals who are typically more eyes-focused were less affected by mask use, we analyzed eye movement data of unmasked faces using EMHMM and conducted a 2 (mask condition) by 2 (face race) by 2 (typical eye movement pattern) mixed ANOVAs on social categorization performance and bias, eye movement measures, and ERPs.

### Eye movement data collection

EyeLink Portable Duo (SR Research) with a 1000 Hz sampling rate was used to record eye movements. A chinrest was placed at the 60 cm distance from the screen to minimize head movements. Pupil and corneal reflection tracking mode and EyeLink default settings for cognitive research were adopted for data acquisition. The standard 9-point calibration and validation procedures were used before each block and whenever the drift check error was more than 1° of visual angle. Each trial started with a solid circle at the screen center for drift check.

### Eye movement data analysis

**Fig. 1:**
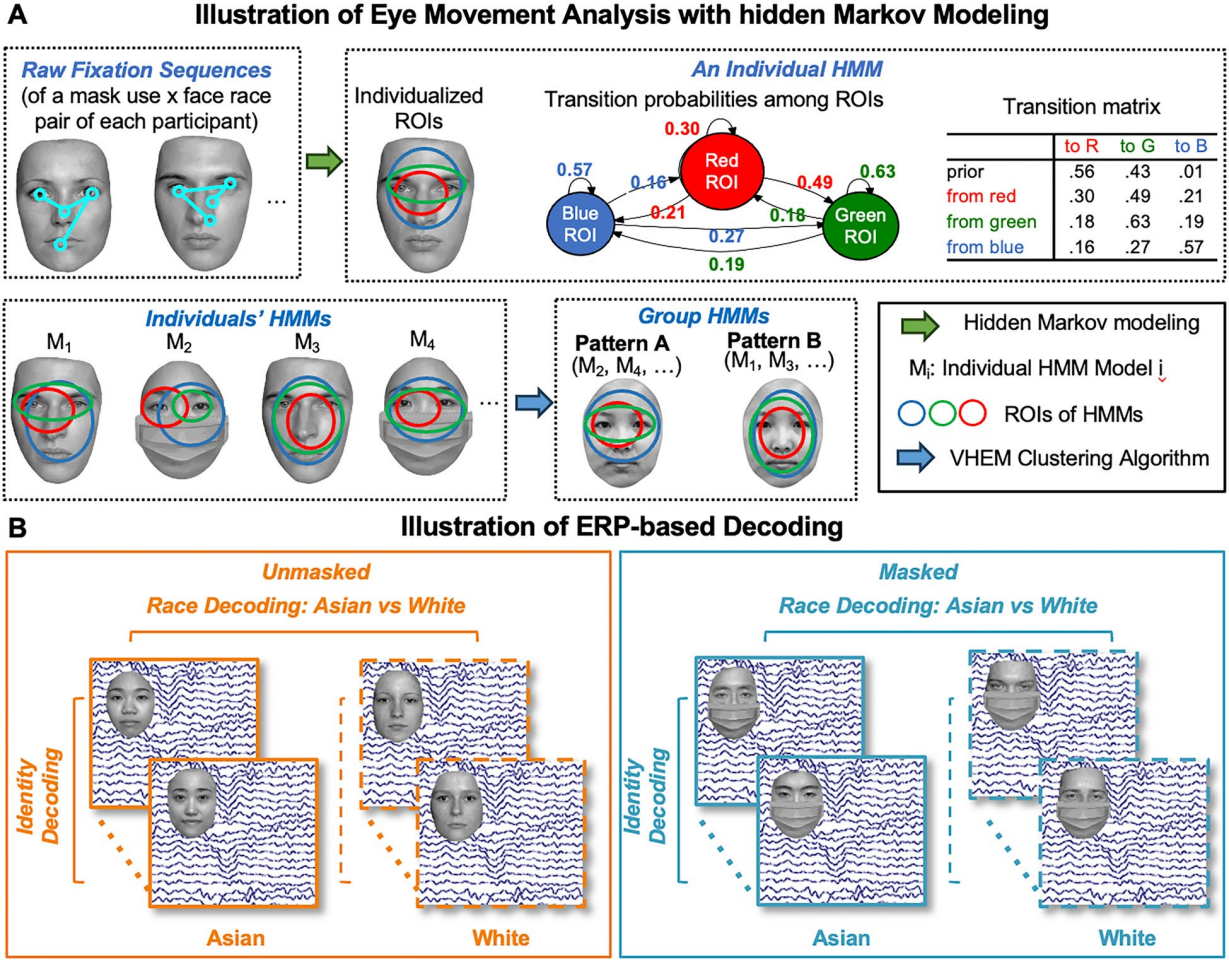
**A** Illustration of Eye Movement Analysis with Hidden Markov Modeling. **B** Illustration of ERP-based Decoding

EMHMM (Chuk et al., 2014; Fig. 1A) was used to analyze eye movement data. A participant’s eye movement data in each of the mask and race condition combinations (i.e., masked Asian, unmasked Asian, masked White, unmasked White) were summarized using a hidden Markov model (HMM, a type of time-series statistical model in machine learning) in terms of ROIs and transition probability among ROIs. The optimal number of ROIs was automatically determined using the variational Bayesian approach (McGrory & Titterington, 2009) from a preset range, which maximizes marginal log-likelihood between an individual’s learned HMM model and their eye fixation sequences across all trials. We preset the range as 1 to 5 ROIs to ensure the algorithm covered variations in individual data with an appropriate level of complexity. For each tested ROI number (K = 1, 2, 3, 4, 5), the variational Bayesian approach learns the best HMM model by estimating all hyperparameters from fixation data. Among these learned models with different ROIs, the one with the highest marginal log-likelihood to the fixation data is selected as the final individual HMM model. The resulting 220 (4 models × 55 participants) individual models were then clustered to discover two representative patterns, pattern A and B, using the variational hierarchical expectation maximization (VHEM) algorithm (Coviello et al., 2014). Following previous studies (e.g., Chuk et al., 2017; Zheng & Hsiao, 2023), we quantified each participant’s eye movement pattern in a condition using A-B scale:

$${\text{A - B}}\;{\text{scale }} = \frac{{\left( {{\text{Loglikelihood A}} - {\text{Loglikelihood B}}} \right)}}{{\left( {\left| {\text{Loglikelihood A }} \right| + { }\left| {\text{Loglikelihood B}} \right|} \right)}}$$

where Loglikelihood A and B represent log-likelihoods of a participant’s eye-movement data being generated by representative models A and B respectively. The log-likelihood measure reflects the similarity of participant’s eye movements towards the representative pattern. A more positive A-B scale indicates a higher similarity toward pattern A.

In addition, we examined participants’ eye movement consistency across trials in different mask conditions using the entropy of the HMMs (entropy is a measure of predictability; higher entropy indicates higher randomness and lower consistency; Cover & Thomas, 2006), which has been used to quantify participants’ eye movement consistency during visual tasks in EMHMM (Qi et al., 2024; Hsiao et al., 2022a).

### EEG data collection

Continuous EEGs were recorded with an eego amplifier and a 64-channel gel-based waveguard cap based on an extended 10–20 layout (ANT Neuro, Enschede, Netherlands). The online sampling rate was 500 Hz, with CPz as the online reference and AFz as the ground electrode. The horizontal electrooculogram (EOG) was recorded from an electrode placed around 1.5 cm to the left external canthus. Electrode impedance was kept below 20 kΩ throughout the recording process to ensure data quality.

### EEG data preprocessing

EEG preprocessing was conducted with customized scripts in MATLAB (MathWorks Inc., Natick, MA, USA), incorporating the EEGLAB (Delorme & Makeig, 2004) and ERPLAB (Lopez-Calderon & Luck, 2014). First, raw EEG signals were downsampled to 250 Hz and bandpass filtered in the frequency range of 0.05–30 Hz using the finite impulse response (FIR) filter implemented in EEGLAB. Line noise at 50 Hz was removed using the CleanLine algorithm (Mullen, 2012). Electrodes for EOG, M1, and M2 were excluded from further analysis. Bad channels were identified and removed through visual inspection before being interpolated. The data were then re-referenced to the common average. To facilitate the independent component analysis (ICA), the EEG data were segmented into [−1000 to 2000 ms] epochs relative to the onset of the face stimuli. Bad epochs were identified through visual inspection and removed from future analyses. The remaining epochs were high-pass filtered at 1 Hz to enhance ICA performance and subjected to ICA. Artifacts caused by eye movements and muscle activity were identified and corrected using visual inspection with the help of the ICLabel plugin (Pion-Tonachini et al., 2019) implemented in EEGLAB. Additional artifacts were automatically identified using a threshold of ± 100 μV. Epochs with incorrect responses (e.g., button presses when a face was present) were excluded. Participants with less than 50% remaining epochs were removed from analysis (n = 3). For the remaining participants, the average number of epochs retained for analysis was 172.90 (SD = 18.93) for masked Asian faces, 176.45 (SD = 18.06) for unmasked Asian faces, 172.55 (SD = 18.39) for masked White faces, and 175.12 (SD = 19.15) for unmasked White faces. These epochs were included in subsequent ERP and ERP-based decoding analyses.

### ERP quantification

For ERP quantifications, continuous EEGs were segmented into [−200 to 1000 ms] epochs and were averaged for ERPs using the −200-0 pre-stimulus as baselines. We analyzed attention-related component P1 (80–120 ms) at a predefined occipital site (O1, O2) (Vizioli et al., 2010), face-sensitive N170 (120–220 ms) at predefined left and right occipital-temporal sites (left: T7, TP6, P7, PO7; right: T8, TP8, P8, PO8) (Chen et al., 2024), and P2 (150–250 ms) at a predefined frontal-central site (F3, F4, Fz, C3, C4, Cz) (Sheng & Han, 2012). These ERP components were selected per prior evidence suggesting they are sensitive to mask use and facial races (e.g., Pesciarelli et al., 2021; Prete et al., 2022; Vizioli et al., 2010).

### ERP-based multivariate decoding

The ERP-based multivariate decoding followed the methods developed by Bae and Luck (2018). Decoding was performed to classify the facial race (Asian vs. White) of the image in both the masked and unmasked conditions, as well as to identify the four distinct identities within each condition (masked Asian, masked White, unmasked Asian, and unmasked White; Fig. 1B). The decoding analysis utilized the scalp-wide distribution of baseline-corrected ERP amplitudes, incorporating data from all channels as features (Bae & Luck, 2018; Lin et al., 2024). Prior to decoding, the epoched data were downsampled to 125 Hz to reduce computational demands and facilitate the analysis.

For race decoding (i.e., Asian vs. White, chance level = 0.50), remaining trials from either Asian or White condition were randomly selected and averaged into 18 ERPs per condition, resulting in a total of 36 ERPs, to increase signal-to-noise ratio. These ERPs were randomly assigned to three folds, each containing 12 ERPs with an equal representation of Asian and White conditions. Decoding was performed on EEG data from 0 to 996 ms relative to face onset, using a 100 ms sliding time window with an 8 ms incremental step. To enhance the signal-to-noise ratio, ERP data were averaged across time points within each window. This produced feature sets consisting of 61 channels for each time window. A threefold cross-validation approach was employed, where two folds were used for training and one for testing, with this process repeated three times. Both training and testing datasets were z-normalized using the mean and standard deviation of the training dataset for each electrode. Subsequently, a support vector machine (SVM) with a one-versus-one approach was used for classification. Classification accuracy was calculated by comparing the predicted labels with the true labels. This procedure was repeated across 10 iterations to ensure reliability, and the accuracy results were averaged across all iterations for subsequent statistical analysis.

For identity decoding (chance level = 0.25), four unique identities were classified within each condition. Due to fewer trials for each identity, all remaining epochs were averaged into 6 ERPs per identity, which were then randomly assigned to 3 folds, each containing eight ERPs. A SVM with a one-versus-all approach was used to classify identities (Bae et al., 2021). Noteworthily, since each identity included two distinct face images (neutral and happy expressions), the decoding reflected identity-level rather than image-level representations.

To determine the statistical significance of the decoding accuracy against chance levels, we employed a cluster-based permutation test (Bae, 2021; Dobs et al., 2019) to correct multiple comparisons. Specifically, clusters of consecutive time points with significant deviations from 0.05 levels were identified, and their t-values were summed to produce a cluster-level t mass. Labels of the decoded conditions were shuffled to create a null distribution of cluster-level t masses from 1000 permutations. Statistical significance was inferred when the true cluster’s t mass exceeded the 95th percentile of the null distribution.

## Results

### The impact of mask on social categorization

**Fig. 2:**
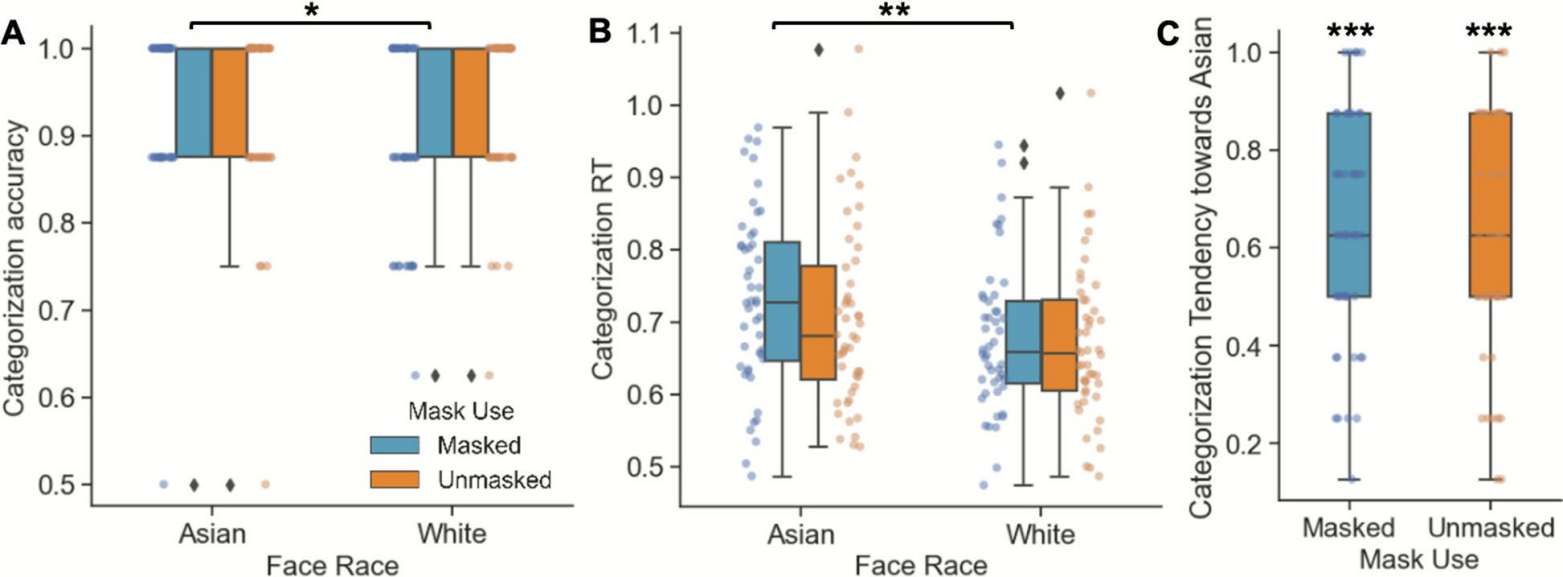
The interaction between mask condition and face race on **A** accuracy and **B** RT in the social categorization task, and the effect of mask condition on **C** the tendency to categorize morphed faces as own race

The effect of mask conditions and face race on social categorization performance and bias was examined using repeated-measures ANOVAs and a paired-sample t-test. For social categorization accuracy, there was no main effect of face race, F(1,47) = 2.43, *p* = .126, mask condition, F(1,47) = 0.91, *p* = .345, or interaction effect, F(1,47) = 3.78, *p* = .058 (Fig. 2A). Based on our hypothesis, we conducted simple t-tests and found that participants had higher categorization accuracy for Asian than White faces under masked condition, t(47) = 2.54, *p* = .015, but not unmasked condition, t(47) < 0.01, *p* = 1.000 (Fig. 2A). This result suggested that mask use made White, but not Asian, faces more difficult to categorize.

For RT, participants responded slower in categorizing Asian than White faces, F(1,47) = 12.64, *p* < .001, η^2^_p_ = 0.212, consistent with the other-race advantage effect in the literature. No effect of mask, F(1,47) = 3.56, *p* = .065, or interaction effect were observed, F(1,47) = 0.931, *p* = .339 (Fig. 2B). This result suggested that mask use did not impact the other-race categorization advantage in RTs. Since the results showed that participants were more accurate while being slower for own-race faces in the masked condition, there may be a speed-accuracy tradeoff. To examine the relationship between accuracy and RT, we performed an exploratory Pearson’s correlation analysis and found that accuracy was not correlated with RT for categorizing masked Asian faces, r(46) = -.020, p = .895, suggesting that the observed other-race advantage effect may not result from speed-accuracy tradeoff.

Regarding categorization bias for biracial morphed faces, morphed faces were more likely to be categorized as Asian/own-race faces under both masked, t(47) = 4.25, *p* < .001, d = 0.61, and unmasked conditions, t(47) = 3.77, *p* < .001, d = 0.55. Moreover, this bias did not differ between masked and unmasked conditions, t(47) = − 0.34, *p* = .737 (Fig. 2C), suggesting that mask use did not modulate own-race biases in categorizing biracial morphed faces.

### The effect of mask use on eye movement pattern

**Fig. 3:**
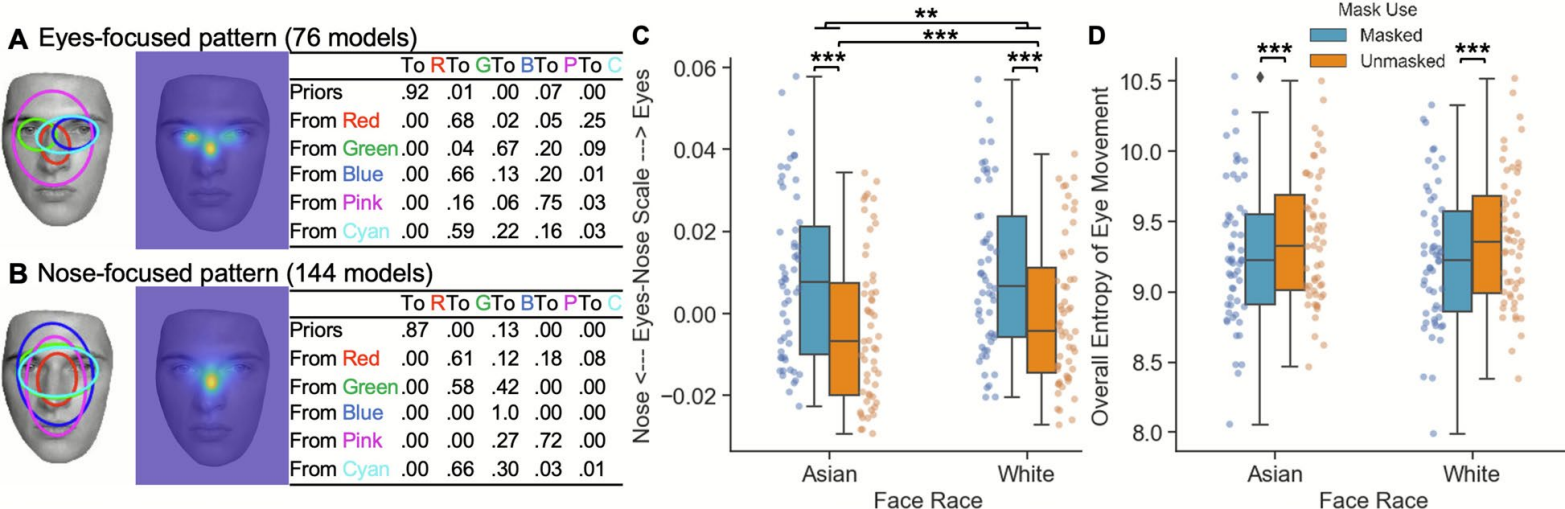
The **A** eyes-focused and **B** nose-focused patterns in free face viewing of identity one-back task. Ellipses show ROIs as 2-D Gaussian emissions. The table shows transition probabilities among the ROIs. Priors show the probabilities that a fixation sequence starts from the ellipse. The image in the middle shows the corresponding heatmap. The effect of mask condition and face race on **C** the eyes-nose scale and **D** the overall entropy of eye movement

We discovered eyes-focused (Fig. 3A) and nose-focused (Fig. 3B) representative patterns, consistent with previous studies (e.g., An & Hsiao, 2021; Hsiao et al., 2021; see Supplementary Section A for more details). Following previous studies, here we refer to A-B scale as Eyes-Nose scale. The effect of mask conditions and face race on eye movement pattern and consistency during face viewing was examined using repeated-measures ANOVAs.

For eyes-nose scale (Fig. 3C), participants were more eyes-focused for White than Asian faces, F(1,54) = 18.14, *p* < .001, η^2^_p_ = 0.251, and for masked than unmasked faces, F(1,54) = 67.96, *p* < .001, η^2^_p_ = 0.557. The race by mask interaction was observed, F(1,54) = 8.23, *p* = .006, η^2^_p_ = 0.132: participants were more eyes-focused when viewing White than Asian faces under unmasked condition, t(54) = − 4.72, *p* < .001, consistent with previous findings (e.g., Fu et al., 2012), but not under masked condition, t(54) = − 2.17, *p* = .144. This result suggested that mask use reduced the other-race effect on eye movement pattern.

For overall entropy of eye movements (Fig. 3D), participants had higher eye movement consistency (lower entropy) for masked than unmasked faces, F(1,54) = 36.35, *p* < .001, η^2^_p_ = 0.402. However, no effect of race, F(1,54) = 0.77, *p* = .384, or interaction effect, F(1,54) = 3.39, *p* = .071, was observed. This suggested that the effect of mask use on eye movement consistency was unaffected by face race.

### The effect of mask use on neural processing and representations of faces

**Fig. 4:**
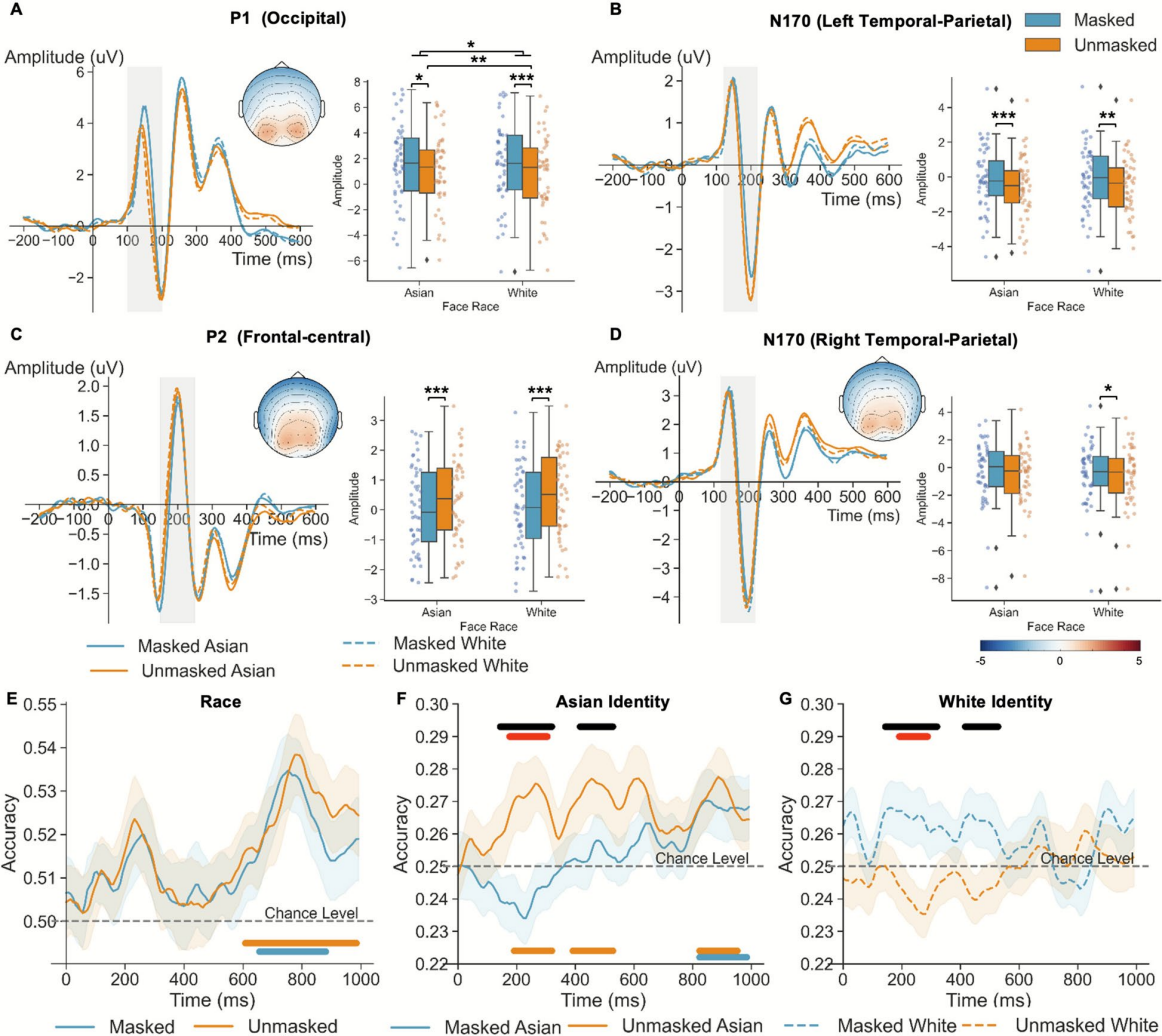
ERP results and ERP-based multivariate decoding accuracy for all subjects. **A** P1 at occipital site, N170 at **B** left and **D** right temporal-parietal site, and **C** P2 at frontal-central site. **E** Race decoding accuracy results. Identities decoding accuracy results for **F** Asian and **G** White faces. The colored horizontal line at the bottom indicated the significant clusters identified by comparing decoding accuracy with chance level for masked (blue) and unmasked (orange), respectively. The black horizontal line at the top indicates the significant cluster for the mask use by race interaction effect. The red horizontal line at the top indicates the significant cluster comparing masked versus unmasked faces. The black dashed line indicates the chance level

The effect of mask conditions and face race on ERP components and decoding accuracy during face viewing was examined. For ERP components, individuals showed more positive P1 amplitude at the occipital site (Fig. 4A) for masked than unmasked faces, F(1,48) = 22.46, *p* < .001, η^2^_p_ = 0.319, and for Asian than White faces, F(1,48) = 8.83, *p* = .005, η^2^_p_ = 0.155. Moreover, we found a significant mask by race interaction, F(1,48) = 4.67, *p* = .036, η^2^_p_ = 0.089: individuals had more positive amplitude for Asian than White faces under unmasked, t(48) = 3.52, p_tukey_ = .005, but not masked condition, t(48) = 0.49, p_tukey_ = 0.961. For face-sensitive N170 at the left and right temporal-parietal sites, unmasked faces elicited more negative amplitude than masked faces (ps < .05; Fig. 4B and D). However, no main effect of race, or their interaction was found (ps > .076). For P2 at the frontal-central site (Fig. 4C), individuals showed more positive amplitude for unmasked than masked faces, F(1,48) = 35.23, p < .001, η^2^_p_ = 0.423, and for White than Asian faces, F(1,48) = 6.96, p = .011, η^2^_p_ = 0.127. No interaction effect was found, F(1,48) = 0.55, *p* = .461. These ERP results suggested that mask use continuously influenced face perception but only modulated the cross-race effect at an early stage (i.e., the P1 component).

Race decoding analyses showed that participants processed race information for both masked (648–880 ms, p_cluster_ = .001, comparing with chance level = 0.25) and unmasked faces (600–984 ms, p_cluster_ < .001), as evident by the significant above chance clusters. However, no difference between mask conditions was observed (Fig. 4E; p_cluster_ > .152). Identity decoding analyses showed that participants differentiated Asian identities for both masked (816–984 ms, p_cluster_ = .017, comparing with chance level = 0.25) and unmasked faces (Fig. 4F; 184–320 ms; 384 – 528 ms; 816–952 ms, p_cluster_s < .001). However, no significant cluster was observed when decoding White identities, regardless of mask conditions (Fig. 4G). An interaction between race and mask from early stage was observed (136–320 ms, p_cluster_ = 0.005; 408–528 ms, p_cluster_ = 0.037, Fig. 4F and G black line): The decoding accuracy was higher for Asian unmasked than Asian masked faces (168–304 ms, p_cluster_ = 0.036, Fig. 4F red line), and for White masked than White unmasked faces (184–288 ms, p_cluster_ = 0.048, Fig. 4G red line). These decoding results suggested that although race processing may be impervious to mask use, identity processing was modulated by mask use.

### Eye-brain-behavior relationship in mask-induced changes

**Fig. 5:**
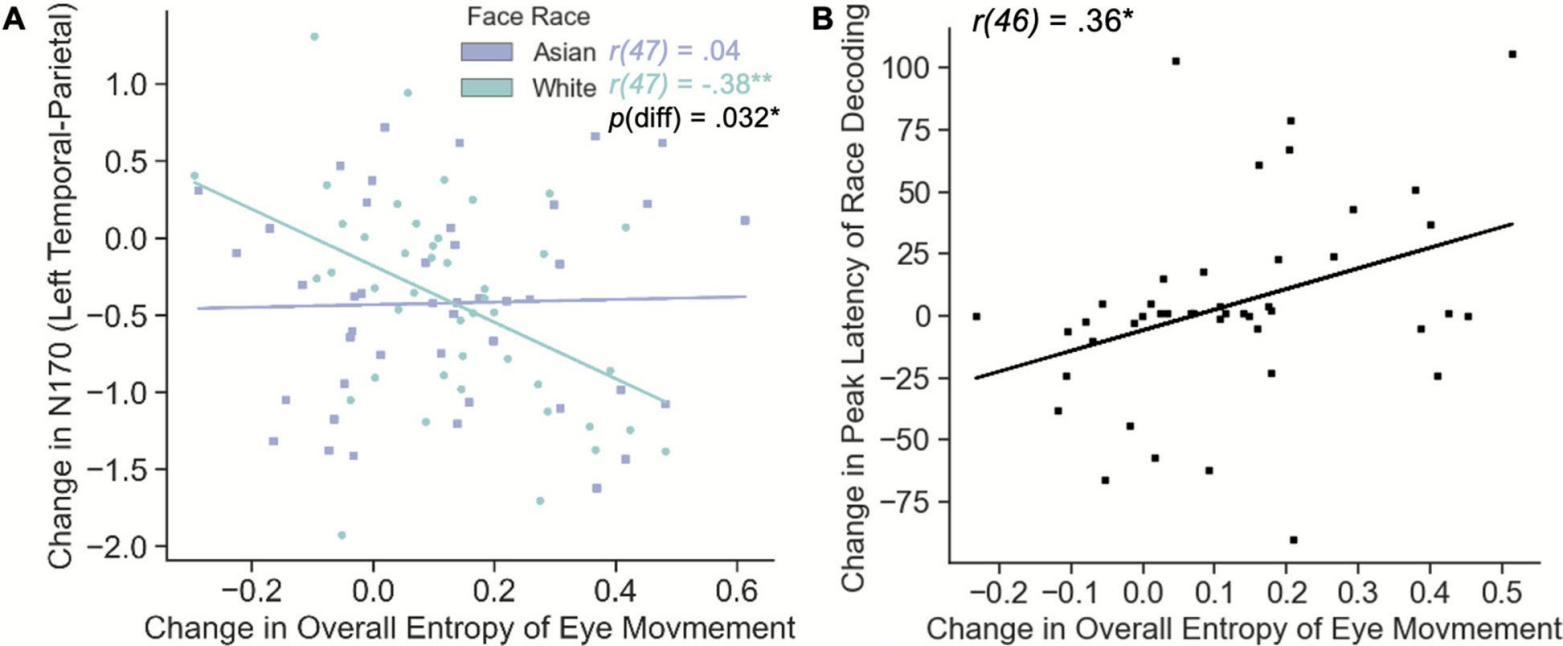
Eye-Brain relationships: The correlation between **A** change in overall entropy of eye movement and change in N170 (left temporal-parietal) for other-race faces, and **B** change in overall entropy of eye movement and change in peak latency of race decoding due to mask use

The relationships among mask-induced changes in behavior, eye movements, neural measures were examined by Pearson’s correlations. The results showed that changes in eye movement and neural measures were not associated with categorization performance changes, ps > .05 (see Supplementary Section B for more details). For eye-brain relationship, the correlational analyses showed that the mask effect in overall entropy (eye movement consistency) was negatively correlated with the mask effect in ERP N170 amplitude in the left temporal-parietal site for White faces, r(47) = − .38, p = .006, but not for Asian faces, r(47) = − .04, *p* = .770 (Fig. 5A). Notably, the correlation coefficients differed significantly, *p* = .042, using the Fisher r-to-z transformation based on a one-tailed test. These results suggested that individuals who developed more consistent visual routine engaged less face processing efforts for other-race faces. This association was not observed in viewing own-race faces, which may result from the perceptual expertise for own-race faces. Also, the mask effect in the overall entropy was associated with the mask effect in peak race decoding latency (Fig. 5B), r(46) = .36, *p* = .011. This indicated that a more consistent eye movement pattern due to mask use was associated with an earlier race decoding peak, consistent with previous research (Liu et al., 2025).

### Typically eyes-focused individuals showed robust neural representations of face race regardless of mask use

**Fig. 6:**
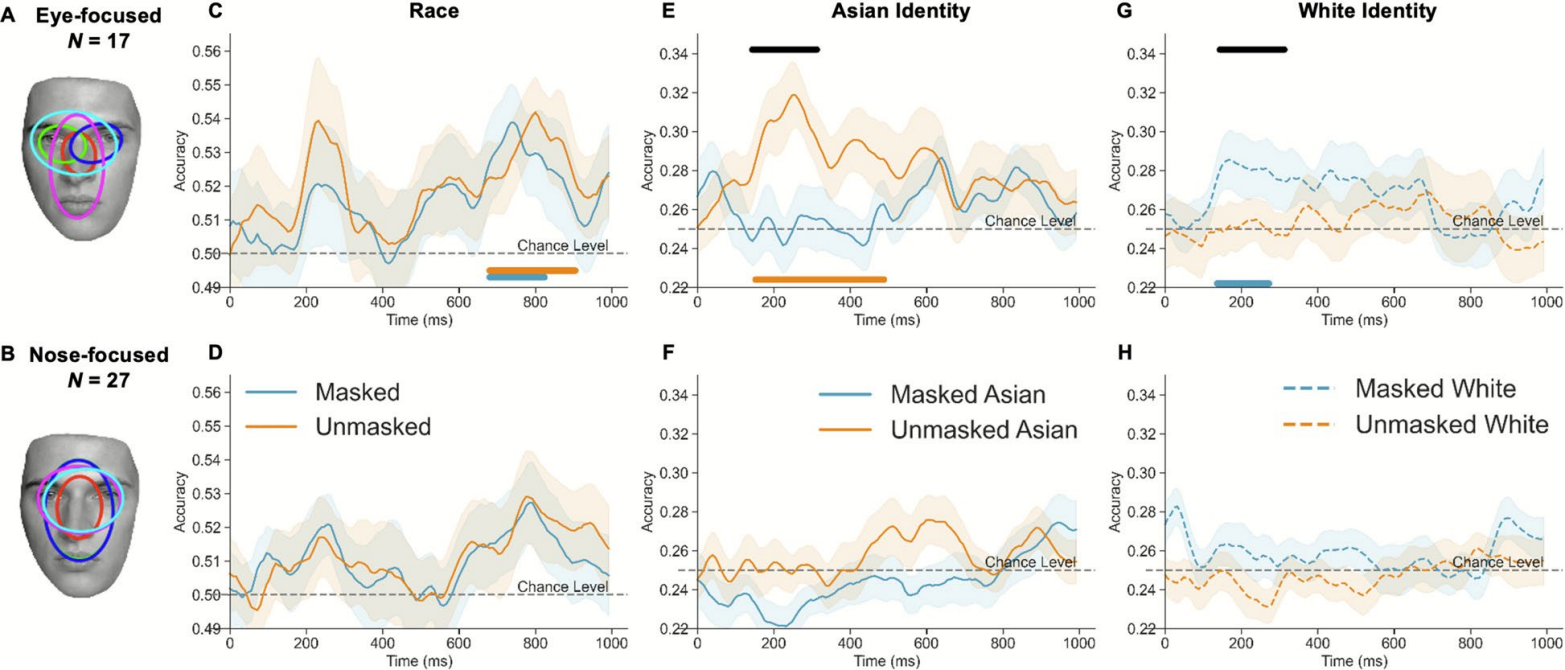
Decoding accuracy was presented for **A** eye-focused and **B** nose-focused participants respectively. The upper row (**C**, **E**, **G**) represents the results from the eye-focused group, and the bottom row (**D**, **F**, **H**) represents the results from the nose-focused group. The colored horizontal line at the bottom of each sub-figure indicated the significant clusters identified by comparing decoding accuracy with chance level for masked (blue) and unmasked (orange) conditions, respectively. The black horizontal line at the top indicates the significant cluster for the mask use by race interaction effect for eye-focused group. The black dashed line indicates the chance level

We also examined whether individuals who typically attend more to the upper half of the unmasked faces (i.e., eye-focused) would be less influenced by mask use. To assess the typical eye movement pattern, EMHMM analysis was performed for eye movement data of unmasked Asian and White faces and clustered individuals into the eye-focused (Fig. 6A) and nose-focused (Fig. 6B) groups (see Supplementary Section C for more details about the two patterns). Only participants who adopted consistent face scanning patterns between Asian and White faces were included in the following analyses. Results showed no interaction effect between typical eye movement pattern and mask condition for categorization accuracy and bias, eye movements and ERPs, suggesting that typical eye movement pattern did not modulate mask effect on these measures (See Supplementary Section D for detailed results).

We then examined the decoding accuracy among eye-focused and nose-focused participants, respectively. 44 participants with valid EEG data adopted consistent patterns, with 17 eye-focused and 27 nose-focused participants. For race decoding accuracy, only eye-focused participants showed above-chance decoding accuracy for both masked (672–824 ms, p_cluster_ = .020) and unmasked faces (672–904 ms, p_cluster_ < .001), with no differences between mask conditions (Fig. 6C). In contrast, no above-chance decoding accuracy was observed for nose-focused participants (masked: p_clusterS_ > .057; unmasked: p_cluster_s > .078; difference: p_cluster_ = .438, Fig. 6D). The results indicate that only eye-focused participants showed a constant neural representation of race even in response to masked faces.

For identity decoding, eye-focused participants showed above-chance Asian identity decoding accuracy for unmasked (144–488 ms, p_cluster_ < .001), but not masked condition (no significant clusters; Fig. 6E). In contrast, these participants showed significant White identity decoding for masked (128–272 ms, p_cluster_ = .003) but not unmasked condition (Fig. 6G). Together, the results indicate that Asian and White faces may induce different face processing strategies, which may lead to different mask-induced changes. In addition, a significant mask by race interaction effect was observed (136–312 ms, p_cluster_ = .005). Nevertheless, post-hoc analyses showed no difference between mask conditions for both Asian (p_cluster_ = 0.075) and White faces (p_cluster_ = .218). Conversely, the nose-focused participants did not show any significant cluster in classifying different identities, or interaction (Fig. 6F and H).

## Discussion

We systematically examined the impact of mask use on the perception and categorization of own- and other-race faces with simultaneous eye-movement and EEG recordings. We found that race modulated the impact of mask use on face perception: mask use impaired participants’ categorization for White faces, but not for own-race Asian faces. For eye-movement patterns, our EMHMM analyses suggest that while participants were more eyes-focused when viewing White than Asian unmasked faces, such differences disappeared for masked faces. For EEGs, mask use modulated face-perception-related ERP components and neural representations of identities for each race respectively, but did not impair the neural representations of races. Furthermore, we found in the mask vs. unmask comparisons, larger reduction in eye movement entropy was associated with a less negative N170 amplitude in viewing other-race faces and with shorter race decoding latencies, suggesting less face processing effort. Notably, individuals who typically adopted eyes-focused patterns showed more robust neural representations of face race regardless of mask use as compared with nose-focused individuals.

Consistent with previous studies (e.g., Anzures et al., 2022; He et al., 2009), race impacts how one perceives faces and the neural processing. Participants showed more positive P1 ERP components when viewing own-race than other-race faces, suggesting people are more sensitive to own-race faces at this early visual processing stage. However, this difference was eliminated when viewing masked faces, suggesting that mask use reduced the other-race effect on P1 activity. This effect of mask use was also observed in eye movement patterns. Consistent with previous findings (Hsiao et al., 2022b; Zheng et al., 2023), mask use directed participants’ attention to the eye region, consequently reduced the other-race effect in eye movement pattern during face reviewing. Nevertheless, mask use did not influence either the other-race advantage in social categorization RT or the social categorization bias in judging ambiguous faces. These results suggested that although mask use significantly modulates the other-race effect in eye movement patterns and neural activities during face viewing, its impact on social categorization seems to be limited. This could be due to that higher-level social categorization judgments stem from stable perceptual representations of own-and other-race faces, which is less influenced by face covering due to mask use. Recent studies in face recognition observed that a larger change towards more eyes-focused and more consistent eye movement pattern was associated with less mask-induced impairment in face recognition (Hsiao et al., 2022b; Zheng et al., 2024). However, here we did not observe the correlation between mask-induced changes in eye movement pattern and consistency and the mask-induced changes in social categorization performance and bias. This difference may result from the inconsistency between tasks used for eye movement measures and behavioral performance. Indeed, eye movement pattern and cross-race effect in neural activities can be task-dependent (e.g., Hsiao et al., 2021; Senholzi & Ito, 2013). The inconsistency between these tasks may have led to different face-viewing strategies, which weakens the relationship between eye movement patterns and behavioral performance.

Regarding the eye-brain relationships, we found an intriguing pattern that mask-induced change in eye movement consistency, rather than eye movement pattern, was associated with mask-induced change in neural representations. More specifically, more mask-induced consistent eye movements were associated with a larger mask-induced decrease in ERP component related to face perception (i.e., N170) only in viewing other-race White faces, but not in viewing own-race Asian faces. This may be due to the perceptual expertise in viewing own-race faces developed over time, and thus, the neural activities of own-race faces were more impervious to mask use and less relevant to eye movement change. For other-race faces, those who can develop consistent visual routines for masked faces may have a better ability to process masked faces. This result is consistent with recent findings where children who are better at developing consistent visual routines (higher eye movement consistency) had better face recognition abilities (Hsiao et al., 2022a). Corroborating this possibility, we found that higher mask-induced consistencies in eye movements were associated with more efficient neural representations of race, as evidenced by earlier race decoding peaks (Liu et al., 2025).

Interestingly, we found that individuals who typically adopted the eye-focused pattern had significant race decoding clusters regardless of mask use, and significant identity decoding clusters for unmasked Asian and masked White faces. In contrast, these significant decoding clusters were not observed among individuals who were typically nose-focused (see also Liu et al., 2025). Previous studies showed that those adopting a more eyes-focused pattern had better face recognition ability (Hsiao et al., 2021, 2022a), since eyes are the most diagnostic features for recognition (e.g., Gosselin & Schyns, 2001). Our results suggested that eyes are critical for race representations (categorization), in addition to identity representations (individuation). Note that eyes-focused participants showed significant identity decoding for unmasked Asian and masked White faces, but not for masked Asian and unmasked White faces. This may be due to greater holistic processing and perceptual expertise for own-race than other-race faces (Michel et al., 2006). Better processing of own-race faces requires more global attention over the whole face, in addition to local attention on the eyes (Xing et al., 2024). As a result, mask use on own-race faces disrupted holistic processing, reducing identity decoding accuracies. Indeed, previous research showed that people were more likely to attend to the eye region for other-race (White) faces and to the nose region for their own-race (Asian) faces (e.g., Hu et al., 2014). Since mask use directed one’s eye movement pattern towards the eyes of the other-race (White) faces for both eyes-focused and nose-focused groups, the advantage of eyes-focused individuals may result from their better processing outcome of eyes with more experience when only the eyes are present.

The current experiment focused on Asian participants’ perceptions of Asian and White faces. However, the impact of mask use may depend on both an individual’s eye movement pattern and the differences in physical features across faces of different races. For example, both White and Asian observers showed greater attention to the nose and mouth region of Black faces as compared with White and Asian faces (Burgund, 2021), and the other-race effect is more salient between Black and White than Asian and White (Zhou et al., 2020). Future work may examine whether these findings can be generalized to non-Asian participants and to faces of other races during social categorization. Also, our research investigated how mask use may modulate race categorization, but it remains unclear whether the current results can be generalized to other social categorizations, such as gender/age categorization and even arbitrary social groups formed using minimal group manipulations (Ng et al., 2020). Future work shall examine these possibilities which will provide more insights on whether the effects are due to physical features or social perception. In addition, there may be a ceiling effect on participants’ behavioral performance in social categorization (92.90%). As a result, the effect of mask use and face race on categorization accuracy may be weakened, which may explain the marginal interaction effect between them in categorization accuracy (*p* = .058). Future work may increase the level of task difficulty to better examine the phenomena. Furthermore, here we investigated eye movements and neural representation in an identity one-back task but behavioral responses in a social categorization task, and these measures can be task-dependent (Hsiao & Chan, 2023; Senholzi & Ito, 2013). Thus, future work may consider examining how eye movements and neural activities influence task performance within the social categorization task. Lastly, our sample was predominantly female due to convenience sampling. An exploratory Welch’s t-test suggested no gender difference in any mask effect measure, except for social categorization RT, *p* = .012. Future research should seek to have a more balanced gender distribution to enhance the generalizability of the current findings.

In conclusion, we systematically examined the effect of mask use on cross-race face perception, and the underlying neurocognitive mechanisms using simultaneous eye-movement and EEG recording. Mask use made participants’ eye movement patterns more eyes-focused and consistent, and reduced other-race effects in both eye movement pattern and early neural representation of faces. Nevertheless, mask-use did not change the bias in social categorization of biracial morphed faces or the other-race advantage in social categorization speed, suggesting that race-based social categorizations primarily rely on eye region and may be impervious to mask use. Together these findings have important implications for cross-race face perception and social categorization: more consistent and more eye-focused visual routines for faces would benefit face processing, even in suboptimal viewing conditions when face features are partially masked.

## Supplementary Information

Additional file1 (DOCX 359 KB)

## Data Availability

Data and materials of this study are available on OSF at https://osf.io/mqjb4/overview?view_only=1379c6ec130241ae9a83c7bb5b77f470.
