# Supplementary material for: The effect of mask use on cross-race face perception: a simultaneous EEG and eye-tracking study
Source: Cogn Res Princ Implic. 2026 Jan 29;11:9. doi: 10.1186/s41235-026-00704-2 (PMC12855653; doi:10.1186/s41235-026-00704-2)
Supplement: Supplementary file 1 — Additional file1 (DOCX 359 KB) [file 41235_2026_704_MOESM1_ESM.docx]

# Supplementary Materials

## Section A. Representative eye movement patterns of the face oddball task

In face viewing, we discovered two representative eye movement patterns as the result of clustering: the eyes-focused (**Figure 3A**) and nose-focused (**Figure 3B**) patterns. This finding was consistent with previous EMHMM studies on face recognition (e.g., Chan et al., 2018; Hsiao et al., 2021a). After the first fixation at the face center/red ROI due to drift correction (92% probability), participants adopting the eyes-focused pattern typically started to fixate on the eye region including left eye/green ROI (68%), right eye/blue ROI (5%) and cyan ROI (25%). After looking at the left eye/green ROI, they had a 4% probability to stay at the green ROI, 67% probability to transit to the right eye/blue ROI, 9% probability to transit to the cyan ROI, and 20% probability to look at other face regions covered by the broad pink ROI. 76 models were assigned to this pattern group. In contrast, participants adopting the nose-focused pattern started at the center of face/red ROI (87%) and mainly look at the face center covered by broad ROIs. 144 models were assigned to this pattern group. The two representative HMMs differed significantly (Chuk et al., 2014): data from those with the eyes-focused pattern were more likely to be generated from the eyes-focused than nose-focused HMM, t(75) = 19.92, p < .001, d = 2.29, and vice versa for the nose-focused group, t(143) = 9.13, p < .001, d = 0.76.

## Section B. Eye-brain-behavior relationship in mask-induced changes

***Eye-Behavior Relationship***

We conducted Pearson’s correlation analyses to examine whether mask-induced changes in eye movement pattern and consistency were associated with mask-induced changes in categorization performance. In contrast to our hypotheses, no such relationship was observed, ps > .05.

***Brain-Behavior Relationship***

We then conducted Pearson’s correlation analyses between mask effects in ERP and EEG decoding measures and the mask effects in categorization performance and bias. We found the P1 mask effect positively correlated with the mask effect in the own-race categorization tendency for both the Asian, r(43) = .30, p = .042, and the White faces, r(43) = .36, p = .015. In addition, the mask effect in N170 at the right temporal-parietal site was positively correlated with the mask effect in own-race categorization bias, for the Asian, r(43) = .34, p = .024, but not the White faces, r(43) = .25, p = .103. However, the mask effect in decoding accuracy/latency was not significantly correlated with the mask effect in categorization performance and bias, ps > .05.

## Section C. Representative eye movement patterns for unmasked faces

In face viewing, we discovered two representative eye movement patterns as the result of clustering for unmasked faces: the eyes-focused and nose-focused (**Figure S1**) patterns. After the first fixation at the face center/red ROI due to drift correction (90% probability), participants adopting the eyes-focused pattern typically started to fixate on the eye region including left eye/green ROI (68%), right eye/blue ROI (23%) and cyan ROI (2%). After looking at the left eye/green ROI, they had a 58% probability to transit to the right eye/blue ROI, 26% probability to transit to the cyan ROI, and 16% probability to look at other face regions covered by the broad pink ROI. 43 models were assigned to this pattern group. In contrast, participants adopting the nose-focused pattern started at the center of face/red ROI (99%) and mainly look at the face center covered by broad ROIs. 67 models were assigned to this pattern group. The two representative HMMs differed significantly: data from those with the eyes-focused pattern were more likely to be generated from the eyes-focused than nose-focused HMM, t(42) = 16.20, p < .001, d = 2.47, and data from those using the nose-focused pattern were more likely to be generated from the nose-focused than the eyes-focused HMM, t(66) = 5.10, p < .001, d = 0.63.


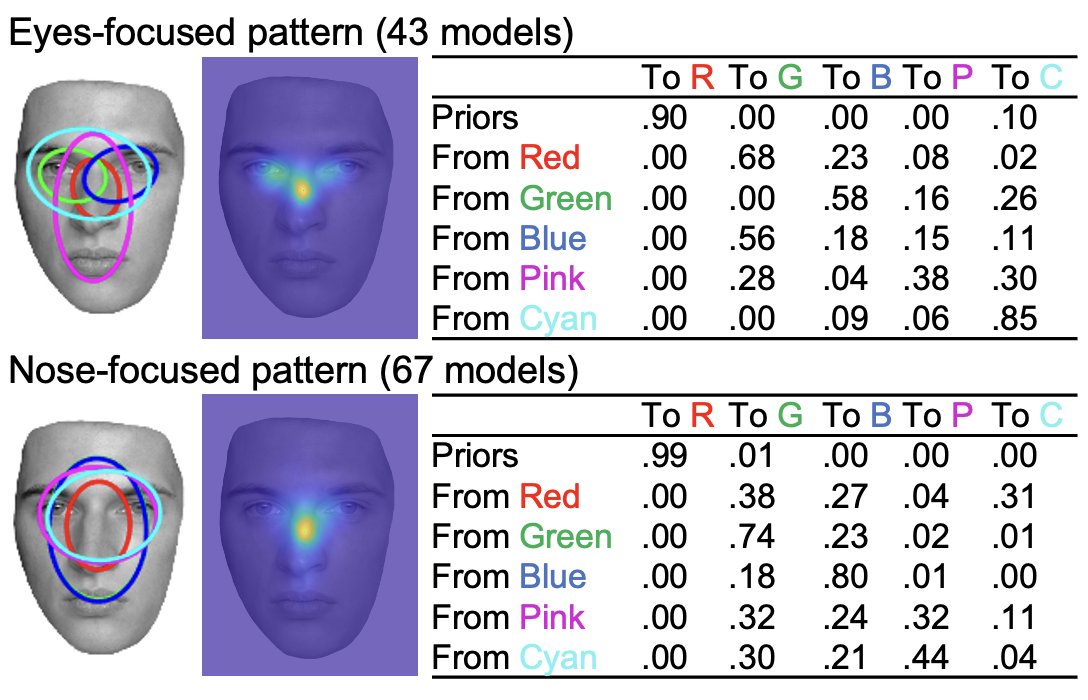


**Figure S1**. The eyes-focused and nose-focused patterns when viewing unmasked (baseline) face stimuli.

## Section D. The impact of typical eye movement pattern on the effect of mask use in cross-race perception

A 2 (mask condition) by 2 (face race) by 2 (typical eye movement pattern) mixed ANOVAs were conducted to examine whether the typical eye movement pattern modulate the effect of mask use on social categorization performance, eye movement pattern, and ERPs^[[1]](#footnote-1)^.

For categorization accuracy, categorization bias, and ERPs during face viewing, no main effect of typical eye movement pattern and its interaction effect with mask condition was observed, ps > .05. This suggested that typical eye movement pattern did not modulate the effect of mask use on race categorization performance and ERPs.

For eye movement pattern, a main effect of typical eye movement pattern was observed, F (1,48) = 127.00, p < .001, η^2^_p_ = 0.726, suggesting that participants who typically adopted an eyes-focused eye movement pattern were more eyes-focused regardless of mask use. However, the pattern group did not interact with face race and mask condition, ps > .05. Similarly, for eye movement consistency, a main effect of typical eye movement pattern was observed, F (1,48) = 14.54, p < .001, η^2^_p_ = 0.232, suggesting that participants who typically adopted eyes-focused pattern had lower overall entropy (higher eye movement consistency) regardless of mask use. However, no interaction with face race and mask condition was observed, ps > .05. This suggested that the typical eye movement pattern did not modulate the effect of mask use on eye movement pattern and consistency during face viewing.

1. Five participants with inconsistent eye movement patterns for Asian and White faces were excluded. As a result, 43 participants with valid social categorization and categorization bias data adopted consistent eye movement patterns, with 17 of them clustered into the eye-focused group and 26 of them clustered into the nose-focused group. In addition, 50 participants with valid eye movement data adopted consistent patterns, with 19 of them clustered into the eye-focused group and 31 of them clustered into the nose-focused group. [↑](#footnote-ref-1)
